# Serum levels of 1,5-anhydroglucitol and 1,5-anhydrofructose-derived advanced glycation end products in patients undergoing hemodialysis

**DOI:** 10.1186/s13098-021-00685-w

**Published:** 2021-08-16

**Authors:** Kenji Tanaka, Akiko Sakasai-Sakai, Yasuki Motomiya, Tatsuo Yoneda, Masayoshi Takeuchi

**Affiliations:** 1 Suiyukai Clinic, Kashihara, Nara 634-0007 Japan; 2 Department of Advanced Medicine, Medical Research Institute, Kanazawa Medical University, Ishikawa, Japan; 3 Unit of Hemodialysis, Nara Medical University, Nara, Japan

**Keywords:** Advanced glycation end products, 1,5-anhydrofructose, 1,5-anhydroglucitol, Hemodialysis, Renal dialysis, Serology

## Abstract

**Background:**

1,5-anhydroglucitol is a reduction product of 1,5-anhydrofructose. Circulating 1,5-anhydroglucitol is usually excreted by the kidneys and is reabsorbed via sodium-glucose co-transporter 4 in the renal tubules. In patients on hemodialysis, serum levels of 1,5-anhydroglucitol have been reported to be low; however, the underlying mechanism remains unclear.

**Methods:**

We measured inter-dialysis changes in the levels of serum 1,5-anhydroglucitol and 1,5-anhydrofructose-derived advanced glycation end products (AGEs) in 78 patients on hemodialysis. Serum levels of 1,5-anhydrofructose-derived AGEs were also determined using a polyclonal antibody.

**Results:**

The serum 1,5-anhydroglucitol level was decreased to as low as 2.0 μg/mL in the regular hemodialysis group; however, we could not verify changes in the serum 1,5-anhydroglucitol level during inter-dialysis days because of undetectable levels in 29 patients. The measured serum level of 1,5-anhydrofructose-derived AGEs was significantly increased in both patient groups. In addition, the 1,5-anhydrofructose-derived AGEs/1,5-anhydroglucitol ratio was higher in patients on hemodialysis than in controls.

**Conclusions:**

Accelerated glycation of 1,5-anhydrofructose is one possible mechanism by which serum 1,5-anhydroglucitol levels are lowered in patients on HD, and we propose that the 1,5-anhydrofructose-derived AGEs/1,5-anhydroglucitol ratio should be measured in clinical settings in which patients have low serum levels of 1,5-AG.

**Supplementary Information:**

The online version contains supplementary material available at 10.1186/s13098-021-00685-w.

## Background

1,5-Anhydroglucitol (1,5-AG) is the exclusive metabolite of 1,5-anhydrofructose (1,5-AF) in humans. It is a reduction product of 1,5-AF, which is continuously produced by ligases derived from glycogen in the liver and other tissues, such as muscle and kidney [1]. The production rate of 1,5-AF is thought to be quite low because serum levels of 1,5-AG have been found to be as low as < 25 μg/mL in healthy individuals, which equates to about one-fortieth of the measured blood glucose level [2, 3]. Circulating 1,5-AG is usually excreted by the kidneys and is reabsorbed via sodium-glucose co-transporter 4 (SGLT4) in the renal proximal tubules [4, 5]. This cotransporter mainly reabsorbs glucose excreted by the glomeruli but can also reabsorb 1,5-AG. Thus, in people with urinary glucose levels exceeding the physiological range, SGLT4 is unable to reabsorb 1,5-AG, which results in decreased serum levels. Considering the physiological function of SGLT4, the serum level of 1,5-AG has been used as a predictive marker of the hyperglycemic state [6–8].

Similarly to 1,5-AG, uric acid (UA) is mostly reabsorbed in the proximal tubules; albeit via another transporter, the urate transporter. A correlation between the serum levels of UA and 1,5-AG has been reported in diabetic patients with early-stage chronic kidney disease (CKD) [9, 10]. In patients with advanced CKD, serum UA levels occasionally increase with disease progression due to a decreased glomerular filtration rate (GFR). Accordingly, patients undergoing hemodialysis (HD) who have lost both excretory and resorptive renal functions may show accumulations of 1,5-AG and UA, since the dialysances of both is almost equivalent. Nevertheless, previous studies have reported that serum 1,5-AG levels are low in HD patients [11, 12]. The discrepancy between serum levels of UA and 1,5-AG is supposedly related to differences in oral intake and metabolic production during inter-dialysis days. However, the metabolic production of 1,5-AG has not yet been adequately studied. Furthermore, the mechanisms by which serum 1,5-AG levels are decreased in HD patients have not been fully studied. In addition, recent clinical studies have indicated a close association between low serum 1,5-AG levels and the development of cardiovascular disease in non-diabetic subjects [2, 13, 14].

Therefore, we measured serum 1,5-AG levels in 78 patients undergoing HD. In 29 HD patients, changes in serum 1,5-AG levels were also calculated during inter-dialysis days in order to determine the metabolic production of 1,5-AG. Furthermore, we measured serum levels of 1,5-AF-derived advanced glycation end products (AF-AGEs), which are novel, exclusive byproducts of 1,5-AF, such as 1,5-AG, using a specific antibody for AF-AGEs [1]. This study was the first to investigate AF-AGEs in a clinical setting in patients before and after HD, as well as in patients with diabetes mellitus.

## Methods

This clinical study involved two protocols: one with HD on the first dialysis day of the week and another with HD on the second and third dialysis days of the week. The latter represents a protocol with an inter-dialysis day between two consecutive HD treatments.

### Subjects

In total, 78 patients (34 women and 44 men) on HD at Suiyukai Clinic were selected as the subjects, with 30 healthy individuals chosen as controls for this study. All patients provided informed consent for participation in our study and underwent HD three times per week. Additionally, 32 diabetic patients (22 patients on regular HD and 10 patients on on-line hemodiafiltration [HDF]) were included. Acutely ill patients were excluded from the study.

### Blood sampling

Blood samples were obtained from arterial sites in an HD circuit at the start and end of HD, after which samples were placed in ethylenediaminetetraacetic acid containers. For niacin testing, blood samples were placed in a specific container.

### Analytical methods

We measured levels of various chemicals, including glycoalbumin, using an autoanalyzer at the laboratory of FALCO Biosystems (Kyoto, Japan). Serum 1,5-AG levels were measured using an enzymatic method at the FALCO laboratory (reference value: higher than 14 μg/mL), and blood concentrations of niacin (nicotinic acid) were measured at the SRL Fukuoka Laboratory (Fukuoka, Japan) (reference range: 4.7–7.9 μg/mL). Serum levels of AF-AGEs were determined using a polyclonal antibody specific for AF-AGEs, as has been reported previously [1].

### Hemodialysis

Thirty-four patients underwent regular HD with a polysulfone membrane, and 14 underwent on-line HDF with a polysulfone membrane. The dialysate used in all patients was an acetate-free bicarbonate fluid. The mean Kt/V value was 1.01 in all patients.

### Statistical analysis

All data are expressed as mean ± standard deviation or n (%). The mean values for 1,5-AG, AF-AGEs, and 1,5-AG/AF-AGEs of the three groups (i.e., regular HD, on-line HDF, and control group) were compared using the Tukey–Kramer's method after ANOVA. Differences in 1,5-AG levels between pre- and post-HD on the second dialysis day and pre-HD on the third dialysis day of the week were assessed using paired *t*-tests. Pearson’s analysis was used to analyze two variables. All analyses were performed using JMP version 9.0.0 (University of California, Merced, California, USA). Statistical significance was set at a *p*-value of < 0.05.

## Results

### Clinical, demographic, and laboratory data

Clinical, demographic, and laboratory data are presented in Table 1. The mean age was 69.8 years in the regular HD group and 70.0 years in the on-line HDF group. The average hemoglobin concentration in the HD group was 10.9 g/dL, which indicated renal anemia; however, these patients were considered well controlled because they were within the reference values of Japanese guidelines for renal anemia. The blood liver transaminase levels were normal. In the HD group, the blood urea nitrogen (65.4 mg/dL) and creatinine (10.6 mg/dL) concentrations were dramatically increased, and the inorganic phosphorus level was also high (5.22 mg/dL). However, there were no contraindications to participation in this study because these patients had end-stage renal disease. On the other hand, all measurement item data in the control group were within reference values. No patients had serum albumin levels < 3.3 g/dL or C-reactive protein levels > 0.5 mg/dL.

**Table 1:** Clinical, demographic, and laboratory data

Variables	Healthy individuals (N = 30) Values, mean ± SD or n/N (%)	Patients on HD (N = 78) Values, mean ± SD or n/N (%)
Age (years)	48.3 ± 8.15	69.9 ± 10.7
Sex (female)	19/30 (63.3%)	34/78 (43.6%)
Body mass index (kg/m^2^)	22.2 ± 3.30	22.8 ± 4.69
Diabetes mellitus	0/30 (0%)	32/78 (41.0%)
Hemoglobin (g/dL)	14.0 ± 1.43	10.9 ± 1.28
Albumin (g/dL)	4.56 ± 0.263	3.62 ± 0.356
Aspartate aminotransferase (IU/L)	23.5 ± 9.78	14.2 ± 7.26
Alanine aminotransferase (IU/L)	21.5 ± 10.3	12.0 ± 6.74
Urea nitrogen (mg/dL)	13.0 ± 3.32	65.4 ± 15.9
Creatinine (mg/dL)	0.726 ± 0.157	10.6 ± 2.97
Uric acid (mg/dL)	5.01 ± 1.40	6.05 ± 1.42
Corrected calcium (mg/dL)^a^	9.35 ± 0.316	9.09 ± 0.568
Inorganic phosphorus (mg/dL)	3.35 ± 0.366	5.22 ± 1.48
Iron (μg/dL)	99.6 ± 33.8	69.8 ± 27.9
C-reactive protein (mg/dL)	0.0267 ± 0.0645	0.281 ± 0.671

^a^Corrected calcium: total serum calcium corrected for serum albumin. HD, hemodialysis

### Serum level of 1,5-AG

**Fig. 1:**
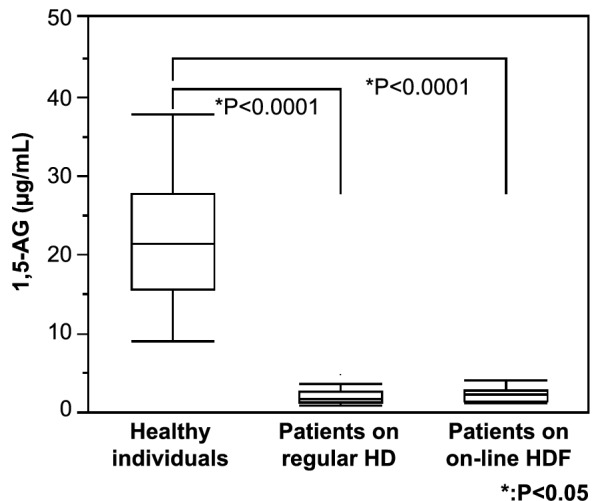
Serum level of 1,5-AG. The serum 1,5-AG level in dialysis patients was measured on the dialysis day at the beginning of the week. In five patients (HD: 3, on-line HDF: 2), the 1,5-AG level was lower than the level allowing for measurement sensitivity, so these patients were excluded. The mean serum concentration of 1,5-AG was 21.3 ± 6.99 μg/mL in the control group. This level was significantly lowered to 1.93 ± 0.823 μg/mL and 2.23 ± 0.817 μg/mL in the HD and HDF groups, respectively. 1,5-AG, 1,5-anhydroglucitol; HD, hemodialysis; HDF, hemodiafiltration

In five HD patients, the levels of 1,5-AG were less than 1.00 μg/mL and not detectable. Therefore, these five patients were excluded, and the 1,5-AG levels of the remaining patients were used in the analysis. The mean serum 1,5-AG level at the start of HD (pre-HD level) on the first dialysis day was 1.93 ± 0.823 μg/mL in the 49 patients on regular HD, 2.23 ± 0.817 μg/mL in the 24 patients on on-line HDF, and 21.3 ± 6.99 μg/mL in the 30 healthy individuals (Fig. 1).

### Change in serum 1,5-AG levels after HD

The change in the serum 1,5-AG level on the second dialysis day of the week was measured in 29 patients on regular HD. The mean pre-HD serum 1,5-AG level was 1.44 μg/mL. After HD, the mean serum 1,5-AG level (post-HD level) was decreased drastically to undetectable levels (< 1.0 μg/mL) in all patients (Table 2).

**Table 2:** Serum levels of 1,5-AG and AF-AGEs at pre-HD and post-HD

Metabolites	Patients undergoing HD (N = 29)
1,5-AG (μg/mL)	
Pre-HD	1.44 ± 0.37
Post-HD	^a^ND
AF-AGEs (U/mL)	
Pre-HD	21.4 ± 9.32
Post-HD	22.7 ± 12.7
Niacin, pre-HD (μg/mL)	5.02 ± 0.71
Glycoalbumin, pre-HD (%)	19.2 ± 8.5

^a^ND, not detectable: less than 1.0 μg/mL. Changes in serum concentrations before and after dialysis were measured in 29 patients who provided consent. The 1,5-AG values after dialysis were lower than the measurement sensitivity in all 29 patients. The mean values for AF-AGEs did not significantly differ at before and after dialysis

### Change in serum 1,5-AG levels between the second and third dialysis days

**Fig. 2:**
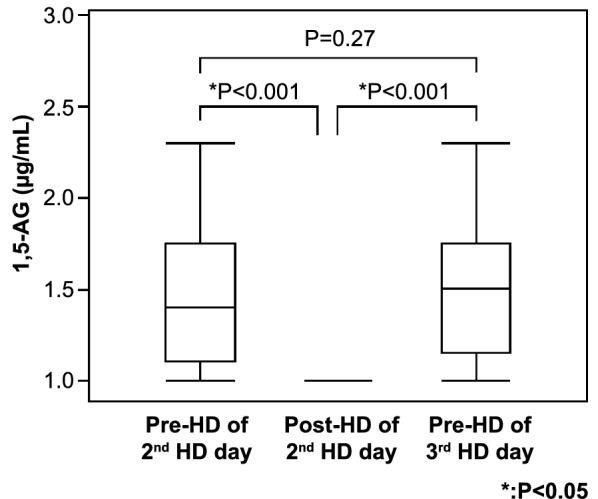
Change in serum 1,5-AG levels between the second and third dialysis days. Using a paired *t*-test analysis, the pre-HD serum 1,5-AG levels from the second and third dialysis days revealed no significant differences. The serum level of 1,5-AG was decreased to an undetectable level after HD and was recovered to almost the pre-dialysis level after 2 days. 1,5-AG, 1,5-anhydroglucitol; HD, hemodialysis

At the start of the third dialysis day, the mean serum pre-HD 1,5-AG level was 1.47 μg/mL, which was approximately the same as the mean pre-HD level on the second dialysis day of the week (1.44 μg/mL) (Fig. 2).

### Niacin level

The blood concentration of niacin was measured in 29 patients undergoing regular HD. The mean blood concentration of niacin was 5.02 ± 0.71 μg/mL (range, 4.0–6.5 μg/mL).

### Serum level of AF-AGEs

**Fig. 3:**
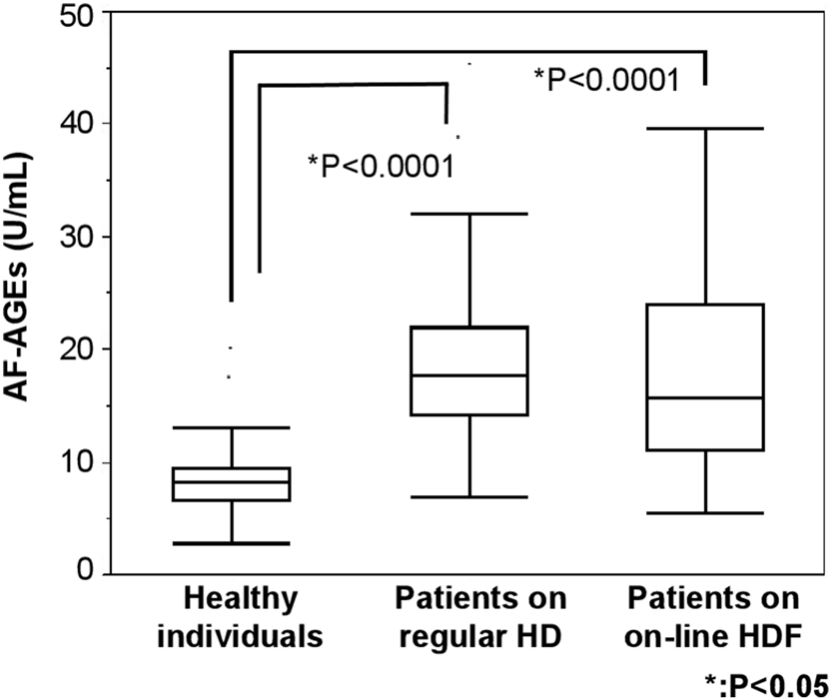
Serum level of AF-AGEs. The serum AF-AGEs level was measured in dialysis patients on the dialysis day at the beginning of the week. The mean serum levels of AF-AGEs were significantly higher in HD patients than in controls. AF-AGEs; 1,5-AF-derived advanced glycation end products; HD, hemodialysis

The mean serum level of AF-AGEs was 18.7 ± 7.77 U/mL in patients on regular HD, 18.1 ± 9.11 U/mL in patients on on-line HDF, and 8.76 ± 3.99 U/mL in healthy persons. The mean serum level of AF-AGEs was significantly higher in the two HD groups than in the control group (P < 0.0001) (Fig. 3).

### Correlation between the levels of serum 1,5-AG and AF-AGEs

**Fig. 4:**
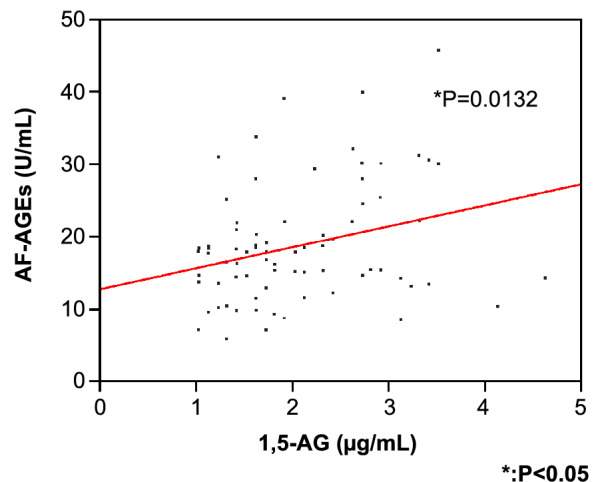
Positive correlation between the serum 1,5-AG and AF-AGEs levels. Our analysis involving 73 patients undergoing HD showed that the serum 1,5-AG level and the AF-AGEs level was positively correlated (R = 0.289, P = 0.0132). 1,5-AG, 1,5-anhydroglucitol; AF-AGEs; 1,5-AF-derived advanced glycation end products; HD, hemodialysis

Both 1,5-AG and AF-AGE are direct end products generated from 1,5-AF. A significant positive correlation was found between the serum levels of 1,5-AG and AF-AGEs in the HD groups (P = 0.0132) (Fig. 4).

### AF-AGEs/1,5-AG ratio

**Fig. 5:**
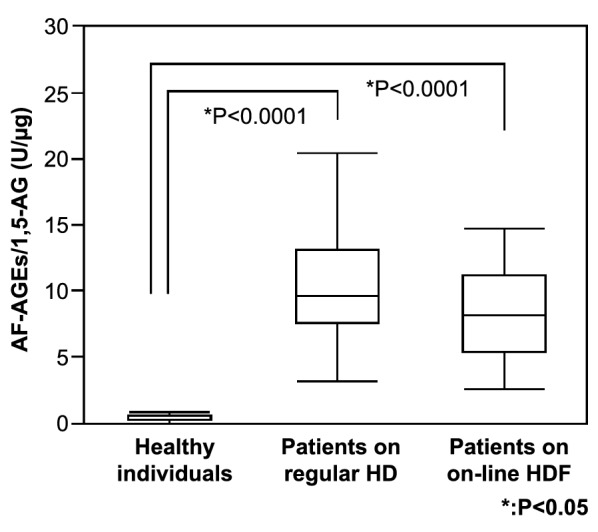
The kinetic ratio of AF-AGEs/1,5-AG. The kinetic ratio of AF-AGEs/1,5-AG was 0.454 ± 0.215 U/μg in the control group (N = 30) and 10.4 ± 4.32 U/μg and 9.08 ± 5.41 U/μg in the HD (N = 49) and HDF groups (N = 24), respectively. 1,5-AG, 1,5-anhydroglucitol; AF-AGEs; 1,5-AF-derived advanced glycation end products; HD, hemodialysis; HDF, hemodiafiltration

1,5-AF is either reduced to 1,5-AG by reductase or forms several glycation products with proteins. Thus, we determined the ratio of AF-AGEs/1,5-AG as the kinetic ratio of both reactions (AF-AGEs/1,5-AF and 1,5-AG/1,5-AF). The mean AF-AGEs/1,5-AG ratio was 10.4 ± 4.32 U/μg in the 49 patients on regular HD, 9.08 ± 5.41 U/μg in the 24 patients on on-line HDF, and 0.454 ± 0.215 U/μg in the 30 healthy persons (Fig. 5). The AF-AGEs/1,5-AG ratio showed an inverse correlation with the serum 1,5-AG level in patients undergoing HD (Additional file 1). In this clinical setting, serum 1,5-AG levels depended on the kinetic ratio of both reactions, as shown in Additional file 1.

### No difference in serum1,5-AG and AF-AGEs levels between diabetic and non-diabetic HD patients

**Fig. 6:**
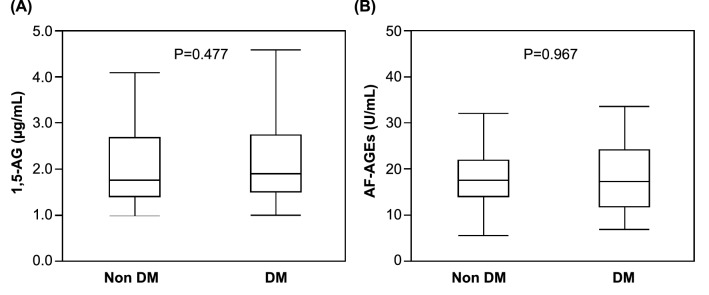
Serum 1,5-AG and AF-AGE levels in diabetic and non-diabetic HD patients. **A** The mean serum level of 1,5-AG was 2.01 ± 0.778 μg/mL in the non-diabetic group (N = 44) and 2.15 ± 0.898 μg/mL in the diabetic group (N = 32). **B** The mean serum level of AF-AGEs was 18.5 ± 7.77 U/mL in the non-diabetic group and 18.4 ± 8.89 U/mL in the diabetic group. There was no significant difference between the two groups. 1,5-AG, 1,5-anhydroglucitol; AF-AGEs; 1,5-AF-derived advanced glycation end products; HD, hemodialysis

The serum 1,5-AG level in patients with diabetes has been reported to be decreased. Thus, the serum levels of 1,5-AG and AF-AGEs were compared between diabetic and non-diabetic patients on HD (Fig. 6). The mean serum values of 1,5-AG and AF-AGEs did not significantly differ between the two patient groups.

### Correlation of the AF-AGEs/1,5-AG ratio with patient age and dialysis duration

**Fig. 7:**
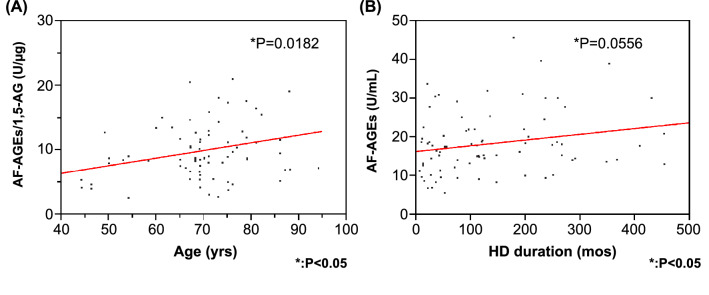
Correlation of the AF-AGEs/1,5-AG ratio with patient age and dialysis duration. **A** The AF-AGEs/1,5-AG ratio showed a significant correlation with age (R = 0.276, P = 0.0182). **B** The serum level of AF-AGEs tended to increase with an increasing dialysis duration (R = 0.218, P = 0.0556). 1,5-AG, 1,5-anhydroglucitol; AF-AGEs; 1,5-AF-derived advanced glycation end products

There was no significant correlation between serum 1,5-AG or AF-AGE levels and age in the dialysis patients (P = 0.476, P = 0.183, respectively), but there was a significant positive correlation between the AF-AGEs/1,5-AG kinetic ratio and age (P = 0.0182). In addition, there was no significant correlation between the serum 1,5-AG level and the dialysis duration (P = 0.151), but the AF-AGE level tended to increase with a longer HD duration (Fig. 7).

## Discussion

**Fig. 8:**
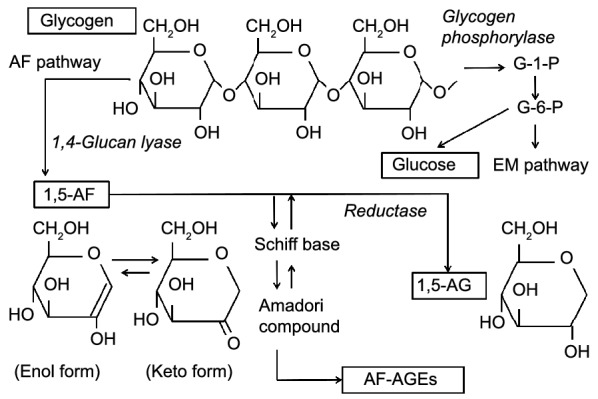
Schema of the glycogen metabolic pathway. 1,5-AF is metabolized to 1,5-AG by AF-specific reductase. AF-AGEs are thought to be novel metabolites derived from 1,5-AF, such as 1,5-AG. 1,5-AF, 1,5-anhydrofructose; 1,5-AG, 1,5-anhydroglucitol; AF-AGEs; 1,5-AF-derived advanced glycation end products; AF pathway, anhydrofructose pathway; EM pathway, Embden-Meyerhof pathway; G-1-P, glucose-1-phosphate; G-6-P, glucose-6-phosphate

1,5-AF and 1,5-AG are metabolites of glycogen in the AF pathway, which is a minor pathway compared with the Embden-Meyerhof pathway (Fig. 8). The serum level of 1,5-AG is a useful biomarker for the hyperglycemic state and has been reported to be low in diabetic patients, patients undergoing HD, and the elderly [6, 11–13]. However, our study unexpectedly showed a nadir value for the serum 1,5-AG level that was clearly lower than what has previously been reported in patients undergoing HD [11, 12].

1,5-AG is considered an exclusive end product of the AF pathway in humans. Therefore, since renal tubular functions are completely disrupted in patients undergoing maintenance HD, the serum 1,5-AG level in HD patients should depend on the production of 1,5-AF, elimination by HD, and food intake. 1,5-AG can move freely through vascular and cellular walls and is distributed evenly, not only in the intravascular and extravascular spaces but also in the intracellular space [15]. Although the 1,5-AG reduction rate with HD has reportedly been as high as > 60% [12], we could not estimate the net amount of 1,5-AG eliminated by HD in this study because of undetectable post-HD serum values. However, in patients with consecutive HD treatments (the second and third dialysis days of the week), the pre-HD level of serum 1,5-AG was nearly the same at two consecutive HD sessions, suggesting that the 1,5-AG eliminated by HD was mostly recovered by metabolic production and oral intake over the interval days; that is, the inter-dialysis days. Assuming that is the case, we can estimate that the daily 1,5-AG production was 15–25 mg/day depending on the body fluid volume, which is much more than the 0.4–0.5 mg/day that was reported by Yamanouchi et al. [16].

Although many foods contain small amounts of 1,5-AG, the contribution of dietary 1,5-AG to the body pool of 1,5-AG is not more than 5 mg/day [16]. Therefore, the recovery of serum 1,5-AG levels during inter-dialysis days likely results from the flow of 1,5-AG from the extravascular pool into the vascular space rather than de novo metabolic production.

1,5-AF is converted exclusively to 1,5-AG by an AF-specific reductase, which requires the coenzyme NADPH, a derivative of niacin [17]. Serum levels of niacin in this study were maintained within the normal range (5.0 ± 0.7 μg/mL), despite abnormally low levels of 1,5-AG. Additionally, serum niacin levels were correlated with neither serum 1,5-AG nor AF-AGE levels. Thus, factors other than niacin depletion are likely to play a role in the 1,5-AG depletion in HD patients.

Recently, an AGE molecule derived from 1,5-AF has been observed in human serum [1]. AF-AGEs are thought to be novel metabolites derived from 1,5-AF, such as 1,5-AG. In body fluids, the enol and keto forms of 1,5-AF exist in equilibrium, and the latter is more likely to form glycation end products with proteins rather than glucose or fructose [1]. The 1,5-AF molecules involved in the formation of AGEs fail to undergo reduction in vivo. We found higher levels of serum AF-AGEs in HD patients than in healthy controls. Moreover, as shown in Additional File 1, the kinetic ratio of both reactions rapidly increased, and the serum 1,5-AG level decreased, which indicates accelerated production of AF-AGEs in HD patients.

Generally, early glycation products, such as Schiff and Amadori compounds, may be produced in amounts several times greater and more rapidly than AGEs. Accordingly, considerable amounts of 1,5-AF molecules are likely involved in the formation of AF-AGEs, avoiding reduction to 1,5-AG. In addition, the 1,5-AF reductase reaction may be slow because of a Km value as high as 1.02 mM [17], which is much greater than the serum 1,5-AG level in humans [18]. As shown in our previous report, 1,5-AF is either metabolized to 1,5-AG or undergoes glycation by intracellular proteins (Fig. 8) [1]. Thus, 1,5-AG production in the body may depend on the kinetics of both reactions, although we could not estimate Km values in this study because of the lack of serum concentrations of 1,5-AF. However, the increased level of AF-AGEs indicates an acceleration of the glycation pathway in HD patients, suggesting that a considerable amount of 1,5-AF is involved in glycation with cellular proteins, leading to decreased 1,5-AG production.

The significant correlation between 1,5-AG and AF-AGEs is a logical finding because both are end products derived from 1,5-AF (Fig. 4). However, the strong reciprocal correlation between the AF-AGEs/1,5-AG ratio and the 1,5-AG level indicates that the glycation process leading to AF-AGE formation results in a decrease in the reduction reaction to 1,5-AG. Therefore, this study strongly suggests that the glycation process of 1,5-AF is one mechanism by which the serum 1,5-AG level is decreased in this patient population. AF-AGEs are undialyzable proteins that elevate serum levels of AF-AGEs and augment the kinetic ratio of AF-AGEs/1,5-AG in HD patients (Fig. 5). However, it may also be worth calculating the AF-AGEs/1,5-AG ratio in other clinical settings in which patients have low serum levels of 1,5-AG.

In a previous study excluding patients with advanced renal dysfunction, it was reported that diagnosing diabetes mellitus based on serum 1,5-AG levels had an inferior ROC compared with fasting blood sugars and that age was associated with serum 1,5-AG levels [19]. In our study involving patients with end-stage renal disease, there were no correlations between the presence or absence of diabetes mellitus or age and the serum 1,5-AG level (P = 0.761, P = 0.610, respectively). In the dialysis patients, it was inferred that a large amount of 1,5-AG was removed, and the loss of 1,5-AG in the body pool influenced the lack of correlation.

Several recent clinical studies have demonstrated that low serum 1,5-AG levels are a strong indicator of cardiovascular disease in patients not undergoing HD, suggesting that 1,5-AG is involved in the development of this disorder [2, 13, 14, 20]. Nevertheless, the mechanisms by which serum 1,5-AG levels are decreased other than poor renal reabsorption have not been investigated.

In healthy individuals, the daily loss of body 1,5-AG by glycation can be recovered by renal reabsorption of 1,5-AG derived from foods; however, this mechanism is lost in HD patients. Accordingly, even if the daily loss of 1,5-AG in the body pool due to the glycation process is negligible, the amount lost over the years could be clinically non-negligible. Therefore, we believe that both prevention of excessive glycation and supplementation of 1,5-AG are important for maintaining normal serum 1,5-AG levels in HD patients.

## Conclusions

Serum 1,5-AG levels were quite low in patients on regular HD, suggesting a significant reduction of 1,5-AG in the body serum pool of HD patients. On the other hand, serum levels of AF-AGEs, a glycation end product derived from 1,5-AF, were significantly increased in HD patients, suggesting a potential mechanism underlying the small pool size of 1,5-AG in HD patients. In addition, the serum AF-AGEs/1,5-AG ratio was extremely elevated in HD patients. Therefore, we believe that the AF-AGEs/1,5-AG ratio should be measured in other clinical settings in which patients have low serum levels of 1,5-AG.

## Supplementary Information

**Additional file 1: Figure S1.** Inverse correlation between AF-AGEs/1,5-AG and 1,5-AG. The kinetic ratio of AF-AGEs/1,5-AG decreased rapidly as serum 1,5-AG levels increased.

## Data Availability

The datasets generated and analyzed during the current study are not publicly available due to privacy and ethical reasons but are available from the corresponding author upon reasonable request.

## Abbreviations

1,5-AF: 1,5-Anhydrofructose
1,5-AG: 1,5-Anhydroglucitol
AF-AGEs: 1,5-AF-derived advanced glycation end products
CKD: Chronic kidney disease
GFR: Glomerular filtration rate
HD: Hemodialysis
HDF: Hemodiafiltration
SGLT4: Sodium-glucose co-transporter 4
UA: Uric acid

## References

[1] Sakasai-Sakai A, Takata T, Suzuki H, Maruyama I, Motomiya Y, Takeuchi M (2019). Immunological evidence for in vivo production of novel advanced glycation end-products from 1,5-anhydro-d-fructose, a glycogen metabolite. Sci Rep..

[2] Selvin E, Warren B, He X, Sacks DB, Saenger AK (2018). Establishment of community-based reference intervals for fructosamine, glycated albumin, and 1,5-Anhydroglucitol. Clin Chem.

[3] Pitkänen E (1982). Serum 1,5-anhydroglucitol in normal subjects and in patients with insulin-dependent diabetes mellitus. Scand J Clin Lab Investig.

[4] Koga M (2014). 1,5-Anhydroglucitol and glycated albumin in glycemia. Adv Clin Chem.

[5] Tazawa S, Yamato T, Fujikura H, Hiratochi M, Itoh F, Tomae M (2005). SLC5A9/SGLT4, a new Na+-dependent glucose transporter, is an essential transporter for mannose, 1,5-anhydro-d-glucitol, and fructose. Life Sci.

[6] Januszewski AS, Karschimkus C, Davis KE, O’Neal D, Ward G, Jenkins AJ (2012). Plasma 1,5 anhydroglucitol levels, a measure of short-term glycaemia: assay assessment and lower levels in diabetic vs non-diabetic subjects. Diabetes Res Clin Pract..

[7] Dungan KM (2008). 1,5-Anhydroglucitol (GlycoMark) as a marker of short-term glycemic control and glycemic excursions. Expert Rev Mol Diagn.

[8] Yamanouchi T, Akanuma Y (1994). Serum 1,5-anhydroglucitol (1,5 AG): new clinical marker for glycemic control. Diabetes Res Clin Pract.

[9] Zhang K, Xue B, Yuan Y, Wang Y (2019). Correlation of serum 1,5-AG with uric acid in type 2 diabetes mellitus with different renal functions. Int J Endocrinol.

[10] Gotoh M, Li C, Yatoh M, Iguchi A, Hirooka Y (2005). Serum uric acid concentrations in type 2 diabetes: its significant relationship to serum 1,5-anhydroglucitol concentrations. Endocr Regul.

[11] Niwa T, Dewald L, Sone J, Miyazaki T, Kajita M (1994). Quantification of serum 1,5-anhydroglucitol in uremic and diabetic patients by liquid chromatography/mass spectrometry. Clin Chem.

[12] Emoto M, Tabata T, Inoue T, Nishizawa Y, Morii H (1992). Plasma 1,5-anhydroglucitol concentration in patients with end-stage renal disease with and without diabetes mellitus. Nephron.

[13] Ouchi S, Shimada K, Miyazaki T, Takahashi S, Sugita Y, Shimizu M (2017). Low 1,5-anhydroglucitol levels are associated with long-term cardiac mortality in acute coronary syndrome patients with hemoglobin A1c levels less than 7.0. Cardiovasc Diabetol..

[14] Ikeda N, Hara H, Hiroi Y, Nakamura M (2016). Impact of serum 1,5-anhydro-d-glucitol level on prediction of major adverse cardiac and cerebrovascular events in non-diabetic patients without coronary artery disease. Atherosclerosis.

[15] Ying L, Ma X, Yin J, Wang Y, He X, Peng J (2018). The metabolism and transport of 1,5-anhydroglucitol in cells. Acta Diabetol.

[16] Yamanouchi T, Tachibana Y, Akanuma H, Minoda S, Shinohara T, Moromizato H (1992). Origin and disposal of 1,5-anhydroglucitol, a major polyol in the human body. Am J Physiol.

[17] Sakuma M, Kubota S, Mouse AKR (2008). Mouse AKR1E1 is an ortholog of pig liver NADPH dependent 1,5-anhydro-D-fructose reductase. Biosci Biotechnol Biochem.

[18] Welter M, Boritza KC, Anghebem-Oliveira MI, Henneberg R, Hauser AB, Rego FGM (2018). Data for serum 1,5 anhydroglucitol concentration in different populations. Data Brief.

[19] Malkan UY, Gunes G, Corakci A (2015). Rational diagnoses of diabetes: the comparison of 1,5-anhydroglucitol with other glycemic markers. Springerplus.

[20] Bai Y, Yang R, Song Y, Wang Y (2019). Serum 1,5-Anhydroglucitol concentrations remain valid as a glycemic control marker in diabetes with earlier chronic kidney disease stages. Exp Clin Endocrinol Diabetes.

