# Supplementary figures and images for: Serum levels of 1,5-anhydroglucitol and 1,5-anhydrofructose-derived advanced glycation end products in patients undergoing hemodialysis
Source: Diabetol Metab Syndr. 2021 Aug 16;13:85. doi: 10.1186/s13098-021-00685-w (PMC8369766; doi:10.1186/s13098-021-00685-w)

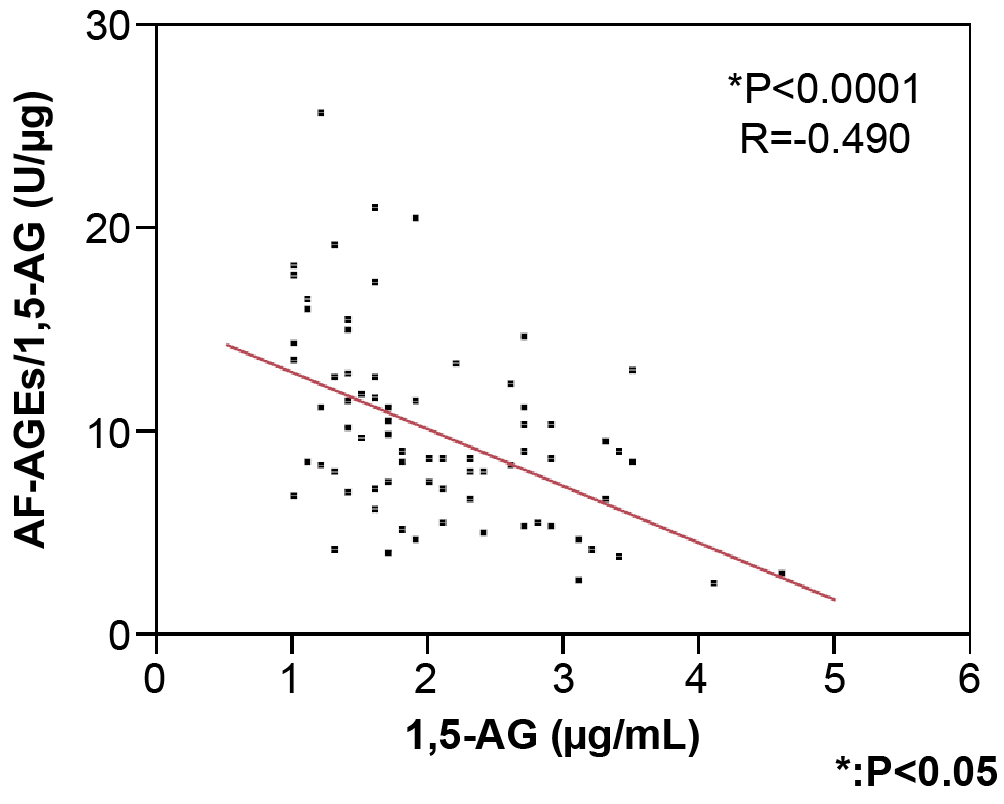

Supplement: Supplementary file 1 — Additional file 1: Figure S1. Inverse correlation between AF-AGEs/1,5-AG and 1,5-AG. The kinetic ratio of AF-AGEs/1,5-AG decreased rapidly as serum 1,5-AG levels increased. [file 13098_2021_685_MOESM1_ESM.jpg]
